# Identifying climate-sensitive infectious diseases in animals and humans in Northern regions

**DOI:** 10.1186/s13028-019-0490-0

**Published:** 2019-11-14

**Authors:** Anna Omazic, Helena Bylund, Sofia Boqvist, Ann Högberg, Christer Björkman, Morten Tryland, Birgitta Evengård, Anders Koch, Camilla Berggren, Alexander Malogolovkin, Denis Kolbasov, Nataly Pavelko, Tomas Thierfelder, Ann Albihn

**Affiliations:** 1 Department of Chemistry, Environment & Feed Hygiene, National Veterinary Institute, Ulls väg 2B, 75189 Uppsala, Sweden; 2 Department of Ecology, Swedish University of Agricultural Sciences, Ulls väg 16, 75007 Uppsala, Sweden; 3 Department of Biomedical Sciences and Veterinary Public Health, Swedish University of Agricultural Sciences, Ulls väg 26, 75007 Uppsala, Sweden; 4 Department of Arctic and Marine Biology, UiT Arctic University of Norway, Biologibygget 2.032, 9019 Tromsø, Norway; 5 Department of Clinical Microbiology, Umeå University, Köksvägen, Infektionskliniken, Universitetssjukhuset, 3187, 90185 Umeå, Sweden; 6 Department of Infectious Diseases, Rigshospitalet University, Blegdamsvej 9, 2100 Copenhagen, Denmark; 7 Department of Infectious Disease Epidemiology and Prevention, Statens Serum Institut, 5 Artillerivej, 2300 Copenhagen, Denmark; 8 University of Greenland, Ilisimatisarfik, Manutooq 1, 3905 Nuuk, Greenland; 9 Department of Energy and Technology, Swedish University of Agricultural Sciences, Lennart Hjelms väg 9, 75007 Uppsala, Sweden; 10 Federal Research Center for Virology and Microbiology (CVM), Academician Bakoulov Street, bldg. 1, Petushki area, Volginsky, Vladimir Region 601125 Russian Federation; 11 Present Address: London School of Hygiene and Tropical Medicine, Keppel Street, WC1E 7HT London, UK

**Keywords:** Ecosystem, Literature search, One health, Transmission, Vector-borne diseases, Zoonoses

## Abstract

**Background:**

General knowledge on climate change effects and adaptation strategies has increased significantly in recent years. However, there is still a substantial information gap regarding the influence of climate change on infectious diseases and how these diseases should be identified. From a One Health perspective, zoonotic infections are of particular concern. The climate in Northern regions is changing faster than the global average. This study sought to identify climate-sensitive infectious diseases (CSIs) of relevance for humans and/or animals living in Northern regions. Inclusion criteria for CSIs were constructed using expert assessments. Based on these principles, 37 potential CSIs relevant for Northern regions were identified. A systematic literature search was performed in three databases using an explicit stepwise approach to determine whether the literature supports selection of these 37 potential CSIs.

**Results:**

In total, 1275 nominated abstracts were read and categorised using predefined criteria. Results showed that arthropod vector-borne diseases in particular are recognised as having potential to expand their distribution towards Northern latitudes and that tick-borne encephalitis and borreliosis, midge-borne bluetongue and the parasitic infection fasciolosis can be classified as climate-sensitive. Many of the other potential CSIs considered are affected by extreme weather events, but could not be clearly classified as climate-sensitive. An additional literature search comparing awareness of climate influences on potential CSIs between 1997–2006 and 2007–2016 showed an increase in the number of papers mentioning effects of climate change.

**Conclusions:**

The four CSIs identified in this study could be targeted in a systematic surveillance programme in Northern regions. It is evident that climate change can affect the epidemiology and geographical range of many infectious diseases, but there were difficulties in identifying additional CSIs, most likely because other factors may be of equal or greater importance. However, climate-ecological dynamics are constantly under change, and therefore diseases may fall in or out of the climate-sensitive definition over time. There is increasing awareness in the literature of the effects of climate change on infectious diseases over time.

## Background

Ongoing climate change is a global concern and the associated warming is most prominent in the far Northern (Arctic) region. This warming of the Arctic profoundly affects its societies, animal populations and environments [1, 2]. The mean global temperature increase since 1880 is 0.85 °C [1], and meta-analyses show that, on average, terrestrial taxa are moving poleward by a median rate of 17 km per decade [3]. Further, the Arctic is currently experiencing the greatest changes in abiotic conditions of any region, as an effect of climate warming [1]. High-latitude ecosystems may be more sensitive to climate-induced changes than their lower-latitude counterparts. A warming climate could change Northern ecosystems rapidly if plant and animal species that are adapted to climate conditions in warmer areas gain the opportunity to extend their geographical distribution into new areas [2].

The changing climate will give opportunities for climate-sensitive infectious diseases (CSIs) to establish or occur sporadically in new areas [4]. Vector-borne diseases are a particular concern in this regard. Arthropod vectors, e.g. ticks, mosquitoes and midges, and reservoir animals, e.g. rodents, birds and wild ungulates, for infectious diseases might also extend their distribution northwards as a result of changes in ecosystems and communities associated with climate warming [5]. The rate of development, persistence and multiplication of most arthropods and microorganisms are directly affected by microclimatic conditions, especially temperature. Warmer temperatures affecting activity and population dynamics of vectors may increase the transmission of pathogens and result in spread to new environments. Climate change affects water availability and humidity in nature, e.g. by changing precipitation patterns and increasing evaporation. An increase in the frequency of extreme weather events (e.g. flooding or drought) that cause an excess or scarcity of drinking water, or natural water in the environment, will affect the epidemiology of some infections and cause epidemics or epizootic outbreaks [6]. Drought and wind can facilitate the spread of soil and dust and thereby also transmission of disease-associated bacteria. *Bacillus anthracis* spores may rise to the surface when heavy rain falls on soil cracked by drying. Conventional methods of storing food and feed may become risky under higher temperatures and/or humidity, since diseases such as botulism and salmonellosis may be favoured by this change.

Today, information regarding the spread of climate-sensitive infections (CSIs) is scarce and in many cases conflicting, e.g. concerning the influence of climate change on their geographical distribution and epidemiology. Despite climate change having an impact on the epidemiology of many infectious diseases, identification of these diseases and determination of the relative importance of climate change for a specific disease on longer timescales are controversial topics. This is partly because many non-climate factors, such as environmental disturbances, land use changes, habitat fragmentation, effects of altered behaviour etc., also affect the incidence of diseases [7, 8]. These factors may have either additive or opposing effects on disease occurrence.

Zoonoses are of special importance in the context of a changing climate. It has been estimated that more than 70% of current human infections are zoonoses [9]. Thus, both animal and human health will most likely be affected by changes in the distribution and virulence of zoonotic pathogens caused by climate change. Further, a population of humans or animals not previously exposed to a particular disease is immunologically naïve, so an outbreak of that disease in a new area will likely have more severe effects.

To gain more knowledge and increase the scope to adjust to a new situation where climate change drives transmission of infectious diseases, a better understanding of the current situation is needed. The first step is to identify CSIs of relevance for humans and animals living at Northern latitudes. Therefore, the aims of the present study were to: (1) identify potential CSIs of relevance for Northern regions; (2) examine whether the available scientific literature supports that the potential CSIs identified are influenced by climate change; and (3) evaluate the effects of climate change on different routes of CSI transmission, based on a systematic literature search.

## Methods

### Selection of climate-sensitive infections

Potential CSIs were selected based on panel discussions among 20 experts representing different fields of expertise, e.g. veterinary and human medicine, animal science, virology, microbiology and ecology. The potential CSIs were chosen among infectious diseases judged to become important in the region reaching from Greenland in the west to Siberia in the east, above latitude 60° N, covering Northern latitude environments from glaciers to tundra and boreal forests.

#### Criteria

To be included as a potential CSI, the infectious agent or disease had to be affected by climate-induced changes in the environment and thereby prone to change its epidemiology, geographical distribution or persistence over time if changes occur. Some opportunistic infections were also considered potential CSIs if they emerge and cause diseases in individuals that are physically stressed, e.g. heat-stressed, due to a changing climate and thereby become immunologically suppressed. Infections meeting the inclusion criteria had to be already present, emerging, expected or recognised as a potential threat in the study region.

#### CSI categories

The potential CSIs were subdivided into five categories based on transmission routes to new individuals, within or between species, in part using the method of McIntyre et al. [10]. These categories were: arthropod vector-borne; food-, feed- and water-borne; soil- and natural water-borne; contact transmission; and wildlife as intermediate host, vector, amplifier or reservoir. Most potential CSIs have several transmission routes, but in this study each infectious disease was placed in just one category, based on the transmission route considered by the experts to be most relevant from a climate change perspective. In addition, wildlife reservoirs are critical for the epidemiology and persistence of certain diseases, so wildlife was established as a separate category in this study.

A potential CSI was included in the arthropod vector-borne category when the microorganism replicates in, and/or is transmitted by, a competent arthropod vector. These CSIs were divided into three subgroups (ticks, midges and mosquitoes) based on their arthropod vector. Transmission of potential CSIs in the food-, feed- and water-borne category may follow consumption of fresh or preserved food by humans, feeding silage or other contaminated feedstuffs to animals or predators consuming prey, etc. Water-borne refers here to spread by freshwater supply/tap water. The soil- and natural water-borne category included potential CSIs transmitted by contaminated surface soil or natural surface water. The contact transmission category included potential CSIs that are transmitted between individuals due to e.g. loss of habitat, restricted pasture or water availability. It also included opportunistic infections already present in a healthy individual that may cause disease due to stress and immunosuppression, or for which increased population density increases the risk of outbreaks. The fifth category of potential CSIs were those that have wildlife as intermediate host, vector, amplifier or reservoir. Wildlife per se are likely to be influenced by climate change and their diseases may spread between individuals by different transmission means. These CSIs are mostly relevant for more than one of the categories outlined above. Wildlife reservoirs and vector animals may consist of many species, but here we divided them into three subgroups: rodents, other mammals, and other animals including invertebrates.

### Literature search

**Fig. 1:**
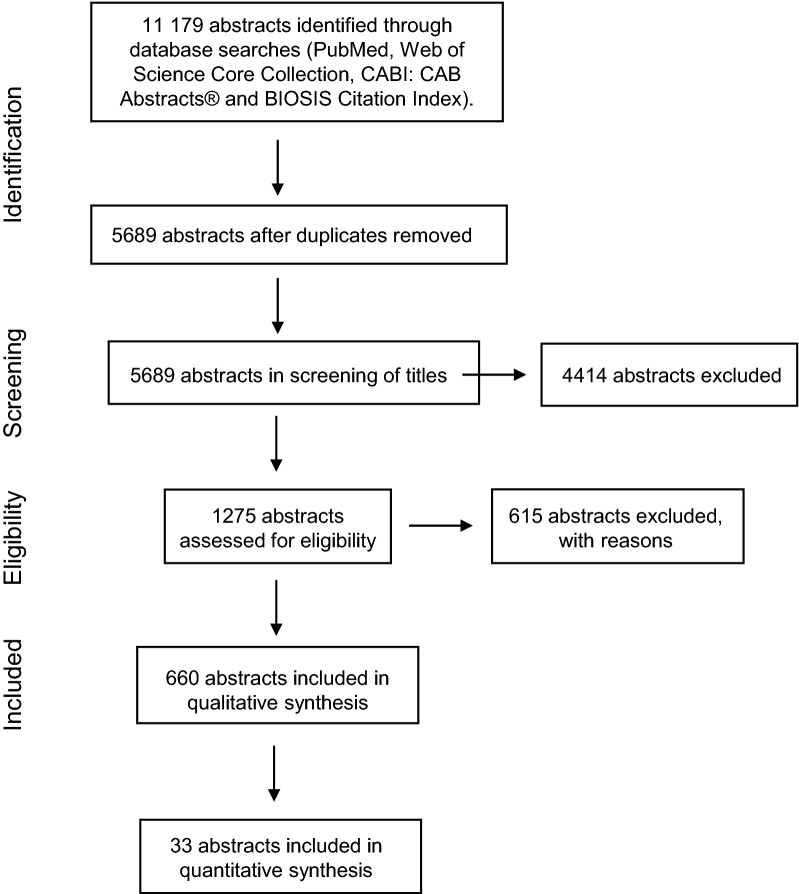
Flowchart of the literature search process. Flowchart of the literature search process used, which was as described in Moher et al. [11], with minor modifications

A systematic literature search was performed as described in Moher et al. [11] with minor modifications. The PRISMA guidelines were used to obtain unbiased results [11]. Keywords and search strings were defined and used for a global search in four databases: PubMed, Web of Science Core Collection, CABI: CAB Abstracts^®^ and BIOSIS Citation Index. Terms used to form search strings is presented in Additional file 1. Only peer-reviewed scientific papers and reviews published from 1997 to October 2017 were selected. All citations were downloaded into the reference manager Endnote (Endnote X7.7) and duplicates were removed, leaving a total of 5689 publications. Screening of titles for relevance reduced the number to 1275 abstracts to be read by the evaluators. A flowchart of the literature search process is presented in Fig. 1.

#### Qualitative synthesis

A total of 12 evaluators from Scandinavia and the Russian Federation with thorough expert knowledge on infectious diseases evaluated the abstracts. Data were extracted from all papers fulfilling the inclusion criteria, which were: abstract in English language, original research on animals and/or humans and studying one of the selected potential CSIs. When the evaluator was uncertain how to rate an abstract, a second evaluator who had been more involved in the design and planning of the literature search read the abstract and made the final decision. Relevant data to identify potential CSIs and describe factors of relevance for this selection of CSIs were extracted by the evaluators using a template created in MS Excel (Table 1). The following variables were included in the template file: publication year, exclusion of an abstract and reason for omission at this stage, characterisation of the disease, geographical area, focus on human and/or animal, the infectious disease studied and category of CSI (as described above). In characterisation of the potential CSIs, the evaluator also had to evaluate whether the information in the abstract suggested that the infection could be classified as climate-sensitive.

**Table 1:** Data extracted from abstracts

Variable	Explanation
Publication year	
Exclusion of an abstract and reason for omission at this stage	Not mentioning one of the selected potential CSIs; not studying animals or humans (only environment)
Characterisation of CSI	The abstract supports that Abiotic: Presence, spread, prevalence or persistence of the CSI is dependent on the ambient temperature, humidity, vegetation cover, surface water or other climate variable Ecosystem: Climate-driven changes in ecosystems or habitats are a driver in the epidemiology of the CSI Vectors and reservoirs: Spread or persistence is dependent on arthropod vectors, intermediate hosts and/or reservoir animals, which in turn are dependent on temperature or other climate variables for their geographical distribution, population density, persistence etc. Opportunistic: Individuals under stress due to environmental and climate conditions are more easily infected with the CSI The infection was classified as climate-sensitive
Geographical area	Country and/or continent
Focus on human and/or animal	
CSI in focus	
Category of CSI	Transmission routes Arthropod vector-borne; Food, feed and water-borne; Soil and natural water-borne; Contact transmission; Wildlife as intermediate host, vector, amplifier or reservoir

Instructions given to readers of abstracts on how to extract relevant information about selected potential CSIs

All abstracts and full papers that classified a potential CSI as climate-sensitive in the qualitative synthesis were read by four evaluators. This was done to further consider and synchronise the assessment of abstracts. Extra care was taken to include only those abstracts that clearly stated climate as a cause of changes in the epidemiology etc. of the disease, and not just single weather events such as heatwaves or flooding.

#### Comparison of awareness of climate influence between two consecutive periods

An additional literature search was performed to determine whether awareness of climate influence increased over time for the potential CSIs. For this purpose, the PubMed database was used to collect available data on all peer-reviewed papers published 1997–2017 and focusing on one or several of the selected potential CSIs. Terms to form search strings for each infectious disease, were used as described above. However, in this additional search, search strings regarding climate and weather events were omitted. All citations were downloaded to Endnote (Endnote X7.7), duplicates were removed, and the remaining references were sorted by year. To examine whether research about potential CSIs had increased over time the difference between the sum of abstracts from the initial systematic literature search and the total number of abstracts found in the additional search was calculated. Two groups were created based on publication year, one consisting of abstracts from 1997 to 2006 and one with abstracts from 2007 to 2016. The year 2017 was omitted from the analysis, since data from the systematic literature search only included abstracts until October 2017. Mean values of the two groups were compared in a two-sample *t* test with equal variance using Stata^®^/IC 15.1 (StataCorp, TX, USA).

## Results

The expert assessments identified 37 potential CSIs, 28 (76%) of which were zoonotic infections (Tables 2 and 3). Table 2 shows the number of abstracts per potential CSI. West Nile fever (n = 100), bluetongue virus (n = 66), borreliosis (n = 42) and tick-borne encephalitis (TBE; n = 33) were the four main CSIs in the arthropod vector-borne category. In the food-, feed- and water-borne category, leptospirosis (n = 100) was the major infectious disease. Anthrax (n = 16) was dominant in the soil- and natural water-borne category. Table 3 shows the dominant diseases in the wildlife category were fasciolosis (n = 45) and hantavirus infection (n = 24), followed by echinococcosis (n = 11), toxoplasmosis (n = 10) and rabies (n = 9).

**Table 2:** Selected potential CSIs distributed into different categories based on mode of transmission

Category		CSI/infectious agent	Zoonotic	No. of abstracts	No. of abstracts that included ≥ 2 CSIs
Arthropod vector-borne	Ticks	Anaplasmosis	Yes	10	17
		Babesiosis	Yes	9	9
		Borreliosis/lyme disease	Yes	42	23
		Tick-borne encephalitis	Yes	33	16
	Midges	Blue tongue disease	No	66	13
		Schmallenberg virus	No	3	3
	Mosquitoes	*Setaria tundra*	No	3	0
		Sindbis fever/Pogosta/Ockelbo	Yes	2	63
		Tularaemia	Yes	10	6
		West Nile Fever	Yes	100	17
Food-, feed- and water-borne		Botulism	Yes	4	1
		Campylobacter infection	Yes	4	18
		Cryptosporidiosis	Yes	7	22
		Leptospirosis	Yes	100	6
		Listeriosis	Yes	0	3
		Salmonellosis	Yes	6	14
		Vtec/EHEC	Yes	0	4
Soil- and natural water-borne		Anthrax	Yes	16	2
		Clostridiosis	Yes	2	2
		*Erysipelothrix rhusiopathiae*	Yes	0	0
		Giardiasis	Yes	2	15
		Q-fever	Yes	2	1
		*Vibrio vulnificus*	No	0	5
Contact transmission		Alphaherpes virus	No	0	0
		Gammaherpes virus	No	0	0
		Necrobacillosis	Yes	0	0
		Parapoxvirus (orf)	Yes	0	1
		Pasteurellosis	No	1	0
		Pestivirus	No	0	0

Selected potential CSIs, divided and subdivided into categories based on mode of transmission to new individuals (within or between species), number of abstracts per CSI and number of abstracts that included more than one CSI

**Table 3:** Selected potential CSIs that have wildlife as intermediate host, vector, amplifier or reservoir

Category	CSI/infectious agent	Zoonotic	No. of abstracts	No. of abstracts that included ≥ 2 CSIs
Wildlife as intermediate host, vector, amplifier or reservoir				
Rodents	Hantavirus	Yes	24	2
Other mammals	Brucellosis	Yes	3	6
	Echinococcosis	Yes	11	2
	Rabies	Yes	9	5
	Toxoplasmosis	Yes	10	2
	Trichinellosis	Yes	1	1
Other animals^a^	*Elaphostrongylus rangiferi*	No	1	0
	Fasciolosis	Yes	45	1

Selected potential CSIs that have wildlife as intermediate host, vector, amplifier or reservoir, here divided into three subgroups: rodents, other mammals, and other animals including invertebrates, following by number of abstracts per CSI and number of abstracts that included more than one CSI

^a^Other animals including invertebrates

**Fig. 2:**
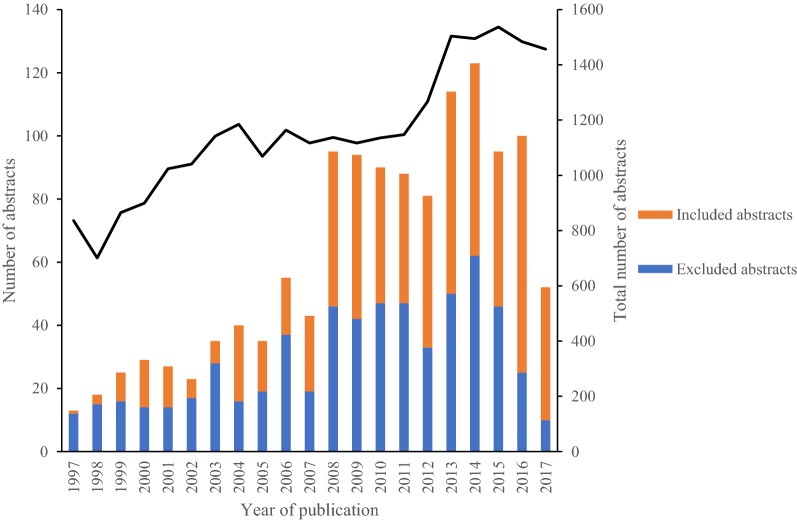
Numbers of abstracts identified. Number of abstracts identified for each year from 1997 to October 2017 (n = 1275), number of abstracts included in the qualitative synthesis (n = 660) and number of abstracts excluded when not relevant, e.g. discussing diagnostic methods and/or not studying animal or human infections and/or not studying animal or humans (only environment) (n = 615). The line shows the total number of abstracts from the additional search for each year 1997–2017 when the climate search strings were left out

Figure 2 illustrates the number of abstracts identified for each year from 1997 until October 2017. In total, 660 abstracts were included in the qualitative synthesis. Of these, 136 abstracts were from 1997 to 2007 and 524 abstracts from 2008 to 2017. Abstracts were excluded when not relevant, e.g. those focusing on diagnostic methods and/or not studying animal or human infections (only environment) (n = 615). Figure 2 also shows the total number of abstracts from the additional search for each year 1997–2017 when the climate search strings were omitted.

Awareness of climate influence between the two periods was tested by comparison of the mean values of the difference between the sum of (included and excluded) abstracts in the initial search and the total number of abstracts in the additional search. This showed that the number of published papers that included climate aspects for the potential CSIs they studied increased (P < 0.01) from the period 1997–2006 (n = 963) to the period 2007–2016 (n = 1202).

**Fig. 3:**
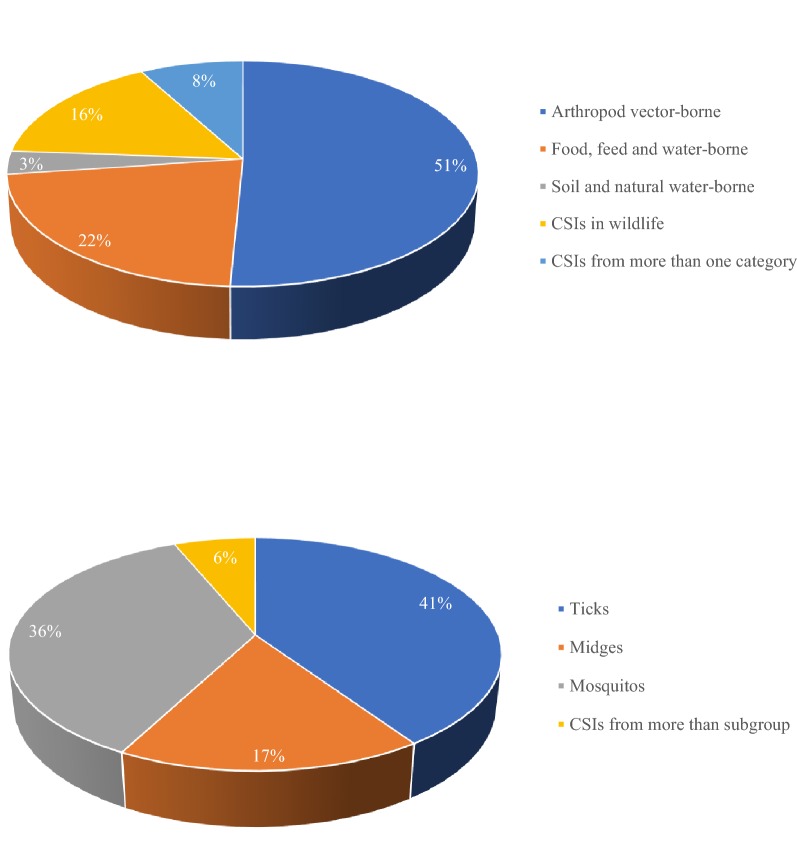
Percentage of abstracts covering each category of climate-sensitive infections. **a** Percentage of evaluated abstracts covering each category of potential CSIs: Arthropod vector-borne; food, feed and water-borne; soil and natural water-borne; contact transmission; and CSIs in wildlife. Abstracts that mentioned CSIs from more than one category were placed in the combined group. **b** Percentage of evaluated abstracts from each of the three subgroups (ticks, midges and mosquitoes) in the arthropod vector-borne category. Abstracts that mentioned CSIs from more than one subgroup were placed in the combined group

**Fig. 4:**
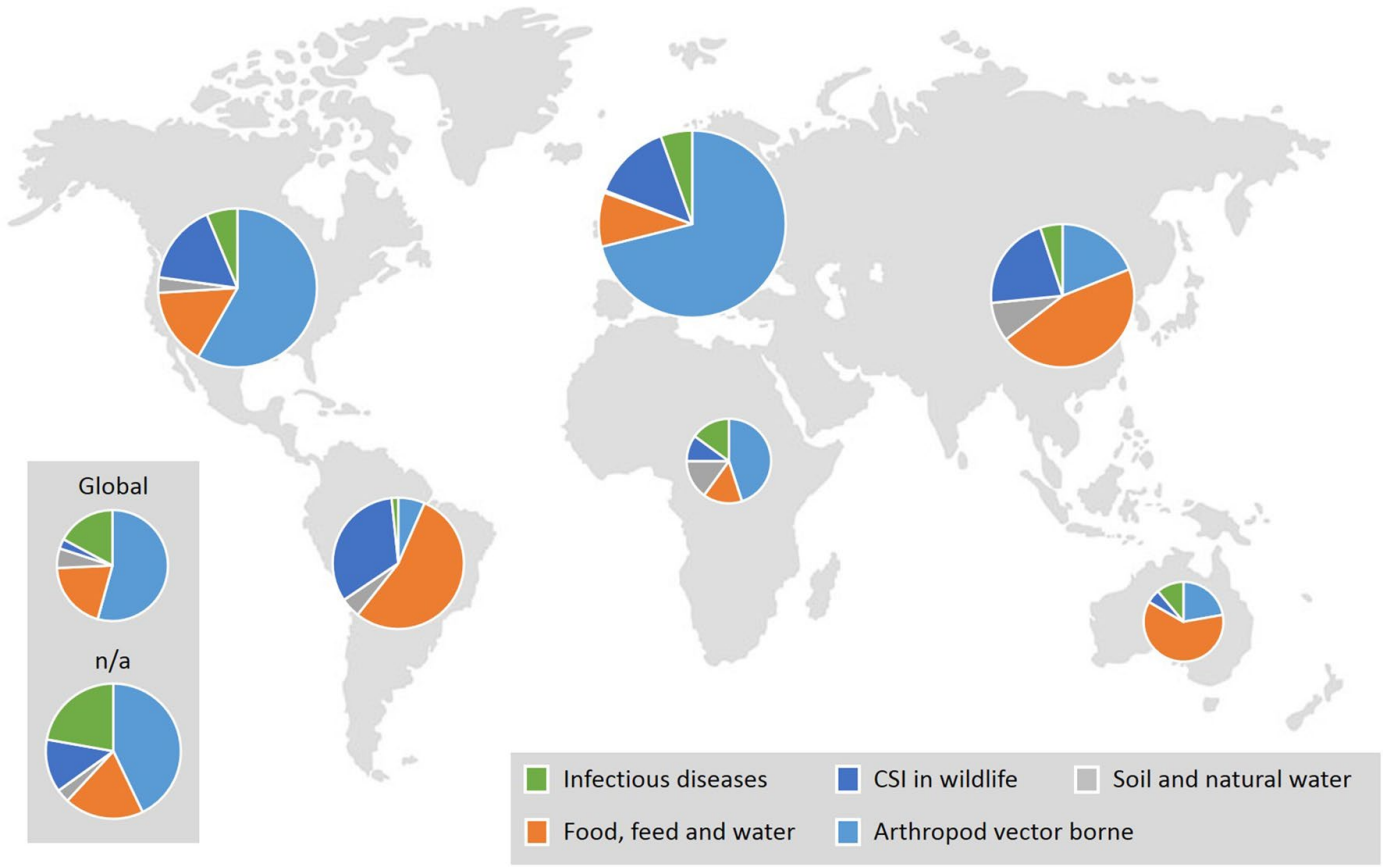
Geographical distribution. Number of abstracts distributed by geographical area for each category of potential CSIs. The size of each circle corresponds relatively on a log-scale to the number of abstracts per geographical area; Europe n = 356, Africa n = 20, Asia n = 80, Australia n = 18, North America n = 127, South America N = 63, global n = 35, n/a n = 35. The contact transmission category had only one abstract and is not shown in the diagram

Around half of the abstracts evaluated (51%) were placed in the arthropod vector-borne category (Fig. 3a). Comparing the distribution of abstracts in the three subgroups in this category (ticks, midges and mosquitoes) showed that ticks were the arthropod vector most often associated with CSIs (41%) (Fig. 3b). The arthropod vector-borne category also contained most abstracts with a European focus (Fig. 4). Further, 54% and 22% of the total number of abstracts evaluated covered tick-borne diseases (TBDs) within Europe and North America, respectively. Moreover, 62% of the abstracts that covered TBDs in North America mentioned West Nile fever. Only two of the abstracts evaluated covered CSIs in the contact transmission category (pasteurellosis and parapoxvirus (orf)). The other selected CSIs in this category (alphaherpes virus infection, gammaherpes virus, necrobacillosis, pestivirus infection) were not mentioned in any of the abstracts evaluated.

Concerning characterisation of CSIs, of all the abstracts evaluated, 30% (n = 199) focused on animals, 44% (n = 287) on humans and 10% (n = 69) on both animals and humans, while 16% (n = 105) did not identify a specific human and/or animal focus. Evaluation of the characterisation of CSIs showed a clear dominance (38%, n = 248) of abstracts in which the presence, spread, prevalence and/or persistence of the CSI was discussed in relation to the ambient temperature, humidity, vegetation cover, surface water or other environmental variable. This was followed by abstracts where the spread or persistence of the CSIs was discussed in relation to arthropod vectors, intermediate hosts and/or reservoir animals (14%, n = 92) and abstracts focusing on climate-driven changes in ecosystems in relation to CSIs (9%, n = 59). Two abstracts included specific comments on the stress on individuals due to environmental and climate conditions facilitating infection and diseases caused by CSI agents. Many abstracts (34%, n = 226) were characterised as falling into more than one group.

The evaluation identified abstracts (5%, n = 33) where the infectious disease was directly affected by climate change and hence classified as a potential CSI. Among these, abstracts relating to the arthropod vector-borne category dominated (transmitted by ticks n = 4; midges n = 1; mosquitoes n = 2). A number of infectious diseases within the food-, feed- and water-borne category (n = 4) and the wildlife category (n = 2) were also classified as potential CSIs. None of the infectious diseases within the soil- and natural water-borne category or the contact transmission category was characterised as a potential CSI.

After the second reading of the 33 abstracts classified as describing potential CSIs, 14 of these were judged not to cover CSIs and those were therefore removed. The synthesis concentrated on 19 full papers published between 2000 and 2017. These results are shown in more detail in Additional file 2. Eight of these full papers were evaluated as presenting climate change as a cause of changed epidemiology etc. and four potential CSIs were stated to be climate-sensitive (borreliosis, TBE, bluetongue and fasciolosis). Full papers that only studied infections affected by short-term, single weather events were not judged to cover CSIs (n = 10), and a full paper not written in English was excluded (n = 1).

## Discussion

It was evident from the review of abstracts and evaluation of full papers in the final synthesis that potential CSIs in the arthropod vector-borne category dominated, supporting earlier findings [10, 12]. The increasing importance of vector-borne diseases (VBD) at Northern latitudes is generally due to expansion of the geographical range for important vector species and their vertebrate hosts. In particular, many publications focus on tick-borne diseases (TBDs) in Europe (Fig. 4). The TBDs listed in Table 2, i.e. anaplasmosis, babesiosis, borreliosis and TBE, were all included in the full paper reading and the final results indicated that borreliosis and TBE can be classified as climate-sensitive. This supports findings in several European studies regarding the influence of climate change, i.e. distribution and expansion to higher altitudes, on TBDs, particularly TBE and borreliosis [13, 14]. However, TBDs illustrate how new information may change opinions on the influence of climate change over time. Dufour et al. [7] decided to exclude TBDs from their list of potential CSIs, while including insect-borne diseases (by mosquitoes and midges), since the participating experts were unable to decide on how ticks would react to climate change.

The midge-borne disease bluetongue was also classified as climate-sensitive, supported by studies showing increased impact of bluetongue as higher temperature opens up new geographical areas for both the vectors and the virus [15–17]. Lastly, fasciolosis, a parasitic infection affecting both wildlife and domesticated animals [18], was classified as climate-sensitive.

The present study included a high proportion (74%) of zoonotic infections. It has been suggested previously that zoonoses are more climate-sensitive than pathogens restricted to humans, due to their wider host and environmental ranges [10]. Climate change is usually not the sole factor causing changes in disease transmission. Changes in the incidence and/or geographical range of CSIs can also arise from interactions between environmental and other factors, e.g. wildlife distribution and changes in land use, that might increase the exposure of local societies and ecosystems. The societal vulnerability may also increase, due to less efficient surveillance and control programmes for CSIs, poor access to veterinary and human healthcare, low education level, inequity and low adaptation to e.g. increasing temperatures. Climate change may increase these and other stressors that affect animal and public health. However, our additional literature search comparing awareness of climate influences in two periods (1997–2006 and 2007–2016) showed that the number of papers studying the effect of climate change on different infections increased significantly (P < 0.01) between the periods.

Characterisation of potential CSIs based on the literature search showed that diseases classified as CSIs are dependent on the ambient temperature, humidity, vegetation cover, surface water or other environmental variables. Arthropod vectors are in general highly impacted by abiotic factors and a changing climate involves changes in temperature and precipitation patterns, which are manifested e.g. in earlier greening and an extended length of vegetation period. Higher temperatures in Northern areas may increase successful overwintering and overall survival of vectors and animal reservoirs, allowing them to expand their distribution range if climate factors have previously been a constraint [2]. High humidity and access to water are crucial for most arthropods, while drought could be detrimental [19].

Leptospirosis was the most dominant disease identified in the food-, feed- and waterborne category. Climate change may alter the habitats and feeding patterns of wildlife species. For domestic animals, new feed crops or changes in feed handling may increase the risk of spread of infectious diseases. Drinking water reservoirs may be contaminated after heavy rain and surface run-off. Flooding and drought may result in water of lower hygienic quality being used.

The number of abstracts on potential CSIs in the soil- and natural water-borne category was limited and, in terms of epidemiology, this is a divergent category of diseases. Spore-forming bacteria, such as *B. anthracis* and *Clostridium* spp., may be spread from soil during extreme weather events, such as flooding, landslides and drought [20]. Most abstracts within this category did not focus on climate change and none of the diseases included was classified as climate-sensitive. However, anthrax received much attention in a study by Walsh et al. [21] on anthrax emergence in the warming North, which identified climate as one of several important factors to include in predictive models. Anthrax spores can be resistant to extreme environmental conditions and can survive for decades in soil [22]. When uncovered, the spores can develop into an infective stage, infecting grazing animals. In one recent example due to the thawing tundra, a study based on DNA sequencing and using protein analysis to categorise permafrost-dwelling microorganisms showed that the release of infective spores from old buried animal carcases caused an outbreak of anthrax in Yamal, Russian Federation, that killed approximately 2500 reindeer and caused many human cases, of which one was fatal [23]. Other diseases in this category may be wind-borne and mainly occur following drought, with q-fever being a relevant example.

Only two of the abstracts evaluated, studying pasteurellosis and parapoxvirus (orf), respectively, were considered to belong to the contact transmission category. One reason for this may be that the other four potential CSIs in this category mainly cause problems in reindeer and other ungulates and may not be much studied with respect to influence of climate change. Opportunistic infections are probably also more relevant for animals, especially wildlife. In domesticated animals, management strategies to reduce heat stress or vaccination may mask the effect of climate change on CSIs. However, actions to mitigate the negative effects of feed shortages, such as corralling and supplementary feeding of semi-domesticated reindeer, could pose an increased risk of spread of infection [24]. In our expert discussions these infections were also characterised as potential CSIs, even if the climate change impact is more indirect and not as obvious as for VBD and wildlife-borne diseases.

When wildlife act as a reservoir of a pathogen or are linked in other ways to the epidemiology of a disease, this often intersects with some or all the transmission categories defined here. Wildlife are dependent on climate variables for their geographical distribution, population dynamics, persistence, migration routes etc. [5]. Results on wildlife as intermediate host, vector, amplifier or reservoir category showed that fasciolosis was dominant and was classified as climate-sensitive. Some of the evidence on fasciolosis found in the literature search was from Mexico, in the South. However, a freshwater snail is always involved in the transmission cycle of fasciolosis and thus wet grassland and mild winters most probably increase the risk of its transmission world-wide. Caminade et al. [25] modelled recent and future climate suitability for fasciolosis in Europe and showed that it increased in central and north-western Europe during the 2000s. This simulated trend is consistent with an observed increase in infected ruminants. The simulation results also showed that recent trends are likely to continue in the future in Northern Europe and will most probably extend the season suitable for development of the parasite in the environment [25]. Hantavirus was also important in this category and highly represented in the study area. The literature search yielded no similar support for five other infectious agents: *Erysipelothrix rhusiopathiae*, *Fusobacterium necrophorum*, and alphaherpes, gammaherpes and pestivirus.

Our literature search in several databases to identify potential CSIs, using a One Health approach, applying a Northern latitude perspective and assessing potential change in awareness of climate change effects on infections in publications over time, showed that VBD, and in particular TBD, poses an increasing threat for high-latitude regions. This supports findings by McIntyre et al. [10], who studied climate influence on animal and human diseases in Europe. In addition, several ambitious efforts have been made to review the impact of climate change on human diseases [12, 26, 27].

In the present analysis, we considered the fact that the word ‘weather’ was used more often than ‘climate’ in most of the abstracts we evaluated and that long-time weather changes are not always referred to as climate change. Thus, the present study provides an indication of several infectious diseases that are most likely to be CSIs and identifies four infectious diseases as climate-sensitive.

The selection of potential CSIs in the present study was subjective and biases might be present. For example, uncommon diseases, present in only one or a few species with restricted expert knowledge, may lead an infectious disease being favoured by one evaluator or rejected by another. A recent disease outbreak and/or increased attention to a disease in the media or in scientific publications may have contributed to bias in inclusion. The search terms used, the exclusion of publications without an English abstract and trends of interest to obtain research funding for a specific pathogen may also have introduced biases. However, these possible biases were likely mitigated by our stepwise approach, i.e. expert discussions, identification of literature, screening of titles, evaluation of abstracts and evaluation of full papers. Further, when organising potential CSIs into different categories, the most general subtype/serotype of the microorganism of a suggested CSI were discussed. However, some CSIs, represented by different subtypes or serotypes, may differ in epidemiology and could therefore be placed in different categories.

The study was based on the literature representing current knowledge (to October 2017) regarding changes in ecosystems and the impact on disease distribution and provides an indication of infections that may be regarded as CSIs. Yet, climate-affected ecological processes are dynamic, and therefore diseases may fall in or out of the climate-sensitive definition over time.

## Conclusions

In the Nordic regions vector-borne diseases, especially tick-borne diseases, are a growing threat. Scientific awareness of the influence of climate change on CSIs has increased over time. From our initial list of 37 potential CSIs, only four diseases (borreliosis, TBE, bluetongue, fasciolosis) could be confirmed as CSIs by the literature search. However, while climate change can affect the epidemiology and geographical range of many infectious diseases, other factors may be of equal or greater importance. The four CSIs identified in this study should be subjected to further research on the effects of climate change on infectious diseases in Northern regions.

## Supplementary information

**Additional file 1.** Terms used to form search strings. Terms used to form search strings in the literature search for studies on potential climate-sensitive infections (CSIs), listed in alphabetical groups.

**Additional file 2.** Support in the literature for diseases classified as climate-sensitive infections (CSIs). Support in the literature for the selected diseases being classified as potential climate-sensitive infections (CSIs). Based on the analysis, tick-borne encephalitis (TBE), borreliosis and bluetongue from the arthropod vector-borne category, and fascioliosis from the wildlife category, were classified as CSIs. Selected potential CSIs from the food-, feed- and water-borne category could not be classified as climate sensitive.

## Data Availability

All data generated or analysed during this study are included in this published article and its supplementary information files.

## Abbreviations

CSI: climate-sensitive infection
TBD: tick-borne diseases
TBE: tick-borne encephalitis
VBD: vector-borne diseases
