# Supplementary material for: Identifying climate-sensitive infectious diseases in animals and humans in Northern regions
Source: Acta Vet Scand. 2019 Nov 14;61:53. doi: 10.1186/s13028-019-0490-0 (PMC6854619; doi:10.1186/s13028-019-0490-0)
Supplement: Supplementary file 1 — Additional file 1. Terms used to form search strings. Terms used to form search strings in the literature search for studies on potential climate-sensitive infections (CSIs), listed in alphabetical groups. [file 13028_2019_490_MOESM1_ESM.pdf]

Additional file 1. Terms used to form search strings

A to B	C to K	L to R	S to W
**alphaherpes***[a] or "alpha herpes"	**campylobacter***	**leptospir*** or "weils disease"	**salmonell***
**anaplasm*** or ehrlichi*	**clostridi***	**listeri***	**schmallenberg** or SBV
anthrax or anthracis	**cryptosporidi***	**necrobacillos*** or "fusobacterium necrophorum"	**setaria** or "filaria* nematode"
**babesios***	**echinococc*** or hydatid*	"**nephropathia epidemica**" or puumalavirus or "puumala virus" or hantavirus	"**sindbis fever**" or pogosta or ockelbo or sindbisvirus or "sindbis virus"
(**bluetongue** or BTV) or "blue tongue"	**elaphostrongylus** or "cerebral nematodiasis" or "brain worm"	**parapoxvirus** or orf or "contagious ecthyma"	"**tick borne encephalitis**" or TBE*
**borreli*** or "lyme disease"	**erysipelothrix** or erysipeloid	**pasteurell***	**toxoplasm***
**botuli***	**fasciol*** or distomatosis or "liver rot"	**pestivirus**	**trichinellos*** or trichuris
**brucellos*** or brucella or "bangs disease"	**gammaherpes*** or "gamma herpes"	**q-fever** or "q fever" or coxiell*	**tularemi*** or francisella or tularens*
	**giardia*** or "beaver fever"	**rabies** or rhabdovirus	**vtec** or **ehec** or enterohemorr$ag* and (“e coli” or “Escherichia coli”)
			"**west nile fever**" or wnf or "west nile virus" or wnv

Search strings

Terms used for each CSI formed one search string, in total 37 CSIs.

((chang* NEAR[b]/2 climat) or “global warming” or “extreme weather” or (chang* AND precipitation) or flood or drought or snow or ((increase* or rising or chang*) AND global AND temperature))

All CSI combined with "OR"

All CSI combined with "AND" ((chang* NEAR/2 climat) or “global warming” or “extreme weather” or (chang* AND precipitation) or flood or drought or snow or ((increase* or rising or chang*) AND global AND temperature))

[a]*=Standard truncation

[b]NEAR is a proximity operator finding records where the terms joined by the operator are within a specified number of words of each other, independent of the order in which the terms occur. NEAR/2 means that the words can be separated by 0, 1 or 2 other words.

Additional file 1. Terms used to form search strings in the literature search for studies on potential climate-sensitive infections (CSIs), listed in alphabetical groups
