# Supplementary material for: Identifying climate-sensitive infectious diseases in animals and humans in Northern regions
Source: Acta Vet Scand. 2019 Nov 14;61:53. doi: 10.1186/s13028-019-0490-0 (PMC6854619; doi:10.1186/s13028-019-0490-0)
Supplement: Supplementary file 2 — Additional file 2. Support in the literature for diseases classified as climate-sensitive infections (CSIs). Support in the literature for the selected diseases being classified as potential climate-sensitive infections (CSIs). Based on the analysis, tick-borne encephalitis (TBE), borreliosis and bluetongue from the arthropod vector-borne category, and fascioliosis from the wildlife category, were classified as CSIs. Selected potential CSIs from the food-, feed- and water-borne category could not be classified as climate sensitive. [file 13028_2019_490_MOESM2_ESM.pdf]

Additional file 2. Support in the literature for diseases classified as climate-sensitive infections (CSIs)

		First read – Abstract		Second read – Full papers	
Category		CSI mentioned in the abstract	No. of abstracts	No. of full papers (ref. no.)	Classified as climate-sensitive
Arthropod vector-borne	Ticks	Anaplasmosis; Borreliosis	1	1 (28)	n/a[a]
		Babesiosis	1	1 (29)	NO
		Tick-borne encephalitis (TBE)	3	3 (14, 30-31)	YES
		TBE; Borreliosis	1	1 (32)	YES
	Midges	Bluetongue	3	3 (15-17)	YES
	Mosquitos	West Nile fever	6	4 (33-36)	Indications[b]
	Tick- and mosquito-borne	Borreliosis; Tularaemia; West Nile fever	1	1 (37)	Indications[c]
Food-, feed- and water-borne		Campylobacter infection	1	0	NO
		Campylobacter infection; Cryptosporidiosis	1	0	NO
		Leptospirosis	6	1 (38)	NO
		Salmonellosis	2	1 (39)	NO
CSI in wildlife					
	Rodents	Hantavirus	2	2 (40-41)	Indications[d]
	Other	Fasciolosis	4	1 (18)	YES
Total			33	19	4

[a]n/a = not applicable. The full paper was not written in English and therefore excluded at this stage.

[b]One article classified West Nile fever as climate-sensitive. Two articles discussed weather extremes effects on West Nile fever and one article was a study about screening of mosquito-borne infections.

[c]There were clear indications in the abstract that the infection discussed was climate-sensitive, but not in the full paper.

[d]One full paper classified Hantavirus as climate-sensitive, and the other paper was not convinced.

Additional file 2. Support in the literature for the selected diseases being classified as potential climate-sensitive infections (CSIs). Based on the analysis, tick-borne encephalitis (TBE), borreliosis and bluetongue from the arthropod vector-borne category, and fascioliosis from the wildlife category, were classified as CSIs. Selected potential CSIs from the food-, feed- and water-borne category could not be classified as climate sensitive.

References

14. Jore S, Viljugrein H, Hofshagen M, Brun-Hansen H, Kristoffersen AB, Nygård K, et al. Multi-source analysis reveals latitudinal and altitudinal shifts in range of Ixodes ricinus at its northern distribution limit. Parasite Vector. 2011; http://www.parasitesandvectors.com/content/4/1/84.
15. Bishop AL, Spohr LJ, Harris AM, Collins D. Factors affecting the distribution of Culicoides spp. (Diptera: Ceratopogonidae) vectors of bluetongue virus (BTV) in Australia. Austral Entomol. 2015;54:385-401.
16. Samy AM, Peterson AT. Climate change influences on the global potential distribution of Bluetongue virus. Plos One. 2016; https://doi.org/10.1371/journal.pone.0150489.
17. Thornley JHM, France J. Blue tongue – A modelling examination of fundamentals – Seasonality and chaos. J Theor Biol. 2016;403:17-29.
18. Cruz-Mendoza I, Quiroz-Romero H, Correa D, Gómez-Espinosa G. Transmission dynamics of Fasciola hepatica in the plateau region of Mexico. Effect of weather and treatment of mammals under current farm management. Vet Parasitol. 2011;175:73-9.
19. Medlock JM, Hansford KM, Bormane A, Derdakova M, Estrada-Peña A, George JC, et al. Driving forces for changes in geographical distribution of Ixodes ricinus ticks in Europe. Parasite Vector. 2013; doi:10.1186/1756-3305-6-1.
28.Sul H, Kim DM. Present state and future of tick-borne infectious diseases in Korea. J Korean Med Assoc. 2017;60:475-83.
29. Pfeifer Barbosa IB, Molnár LĖ, Tavares Dias HL. Determination of the serological prevalence of equine babesiosis by if a test in the state of Pará, Brazil. Rev Bras Parasitol Vet. 2000;9:7-10.
30. Danielová V, Kliegrová S, Daniel, Beneš C. Influence of climate warming on tick-borne encephalitis expansion to higher altitudes over the last decade (1997-2006) in the highland region (Czech Republic). Cent Eur J Public Health. 2008;16:4-11.
31. Daniel M, Kríz B, Danielová V, Valter J, Benes C. Changes of meteorological factors and tick-borne encephalitis incidence in the Czech Republic. Epidemiol Mikrobiol Immunol. 2009;58:179-87.
32. Daniel M, Materna J, Hönig V, Metelka L, Danielová V, Harčarik J, et al. Vertical distribution of the tick Ixodes ricinus and tick-borne pathogens in the northern Moravian mountains correlated with climate warming (jeseníky mts., Czech Republic). Cent Eur J Public Health. 2009;17:139-45.
33. Anyamba A, Small JL, Britch SC, Tucker CJ, Pak EW, Reynolds CA, et al. Recent weather extremes and impact on agricultural production and vector-borne disease outbreak patterns. Plos One. 2014;9:1-9.
34. Rosile PA, Bisesi M. Novel incidences of meteorological drivers of West Nile Virus in Ohio Culex species mosquitoes from 2002-2006 J. Environ Health. 2017;79:16-22.
35. Soverow JE, Wellenius GA, Fisman DN, Mittleman MA. Infectious diseases in a warming world: how weather influenced West Nile virus in the United States (2001-2005). Environ. Health Persp. 2009;117:1049-52.
36. Verna F, Modesto P, Radaelli MC, Francese DR, Monaci E, Desiato R, et al. Control of mosquito-borne diseases in northwestern Italy: preparedness from one season to the next. Vector-borne Zoonot. 2017;17:331-9.
37. Andersen LK, Davis MDP. Climate change and the epidemiology of selected tick-borne and mosquito-borne diseases: Update from the international society of dermatology climate change task force. Int J Dermatol. 2017;56:252-9.
38. Ileana G. Leptospire prevalence and distribution in canine population from Galati county. Lucrări Ştiinţifice. 2011;54:104-8.
39. Milazzo A, Giles LC, Zhang Y, Koehler AP, Hiller JE. Heatwaves differentially affect risk of Salmonella serotypes. J Infection. 2016;73:231-40.
40. Clement J, Vercauteren J, Verstraeten WW, Ducoffre G, Barrios JM, Vandamme AM, et al. Relating increasing hantavirus incidences to the changing climate: the mast connection. Int J Health Geogr. 2009;8:1-11.
41. Kuenzi AJ, Morrison ML, Madhav NK, Mills JN. Brush mouse (Peromyscus boylii) population dynamics and hantavirus infection during a warm, drought period in southern Arizona. J Wildlife Dis. 2007;43:675-83.
